# Identification of a neuron-specific ferroptosis in the neurodegenerative mucopolysaccharidosis III model

**DOI:** 10.3389/fmolb.2025.1476513

**Published:** 2025-03-18

**Authors:** Mathilde Larribau, Myriam Rouahi, Christophe Santiago, Jérôme Ausseil, Zoubida Karim

**Affiliations:** ^1^ University of Toulouse, INFINITY, INSERM UMR1291, CNRS UMR5051, Toulouse, France; ^2^ Laboratory of Biochemistry and Molecular Biology, Centre Hospitalo-Universitaire (CHU) Toulouse, Toulouse, France

**Keywords:** MPSIIIB, iron accumulation, ferroptosis, oxidative stress, neuron

## Abstract

Sanfilippo syndrome (MPSIII) is a neurodegenerative disorder caused by enzyme deficiencies, leading to the toxic accumulation of heparan sulfate oligosaccharides in the brain. Emerging evidence suggests that ferroptosis, an iron-dependent form of cell death, contribute to neurodegeneration. To investigate ferroptosis in MPSIIIB, we examined its regulatory mechanisms and markers in MPSIIIB brains. Our results showed elevated iron levels, decreased mRNA expression of TFR1 and ZIP14 (involved in iron uptake) at 9 months of age, and increased protein levels of FTH (which stores intracellular iron) in MPSIIIB brains, indicating a potential link to ferroptosis. We also observed diminished levels of ferroptosis-neutralizing proteins (xc-/GPX4), while the protective pathway (Keap1-Nrf2) was activated. Oxidative homeostasis disruption was revealed by increased expression of genes encoding SOD2, SIRT3, iNOS, and nNOS enzymes. Increased expression of lipid peroxidation genes (ascl4 and lpcat3) further supported ferroptosis involvement. Furthermore, we analyzed protein abundance and brain immunostaining of the iron exporter FPN. Despite its high expression levels, this protein appeared misfolded and was insufficiently targeted to cellular plasma membrane, which might contribute to cellular iron retention. The co-localization of FPN with NeuN, a marker of neurons, demonstrates that only neurons are affected by this targeting defect, suggesting neuronal ferroptosis specifically in MPSIIIB. Overall, our findings evidenced of the involvement of ferroptosis in MPSIIIB pathogenesis, highlighting dysregulation in iron homeostasis, antioxidant defenses, and lipid peroxidation as key features of the disease.

## 1 Introduction

Sanfilippo Syndrome, also known as mucopolysaccharidosis type III (MPS III), is a rare genetic disorder characterized by the deficiency of specific enzymes required for the degradation pathway of heparan sulfate, a type of glycosaminoglycan (GAG). Theses enzyme deficiencies lead to the accumulation of heparan sulfate oligosaccharides (HSO) in various tissues, particularly in the brain, resulting in cellular dysfunction and progressive neurodegeneration (Neufeld and Muenzer, 2001). The four distinct subtypes of MPSIII (A, B, C and D, each corresponding to a specific enzyme deficiency) exhibited common features including developmental regression, hyperactivity, sleep disorders and distinct somatic abnormalities.

The links between the accumulation of HSO and the neurodegeneration observed in patients have been extensively explored over the past few years. Neuroinflammation have been identified as one of the hallmarks of MPS III pathogenesis in both patients and the mouse models. In the mouse brain, microglia and astrocytes are early responders, and microgliosis has also been described (Ohmi et al., 2003; DiRosario et al., 2009; Holley et al., 2018). HSO has been found to act as a ligand for Toll-like receptor 4 (TLR4) and mediates the activation of microglia as early as 10 days in MPS IIIB mice (Ausseil et al., 2008), suggesting that HSO primed microglia early in the course of the disease. We also demonstrated that activated microglia can impact neurons via inflammatory cytokines (Puy et al., 2018); however, the pathogenic mechanisms driving the neurodegenerative process in neurons remain to be identified.

Ferroptosis, prevented by iron chelation, is a form of regulated cell death characterized by the iron-dependent accumulation of lipid peroxides within the cell membrane (Dixon et al., 2012). This distinctive mode of cell death has received significant attention in recent years due to its implications in various neurodegenerative diseases, including Parkinson’s disease, Alzheimer’s disease, and multiple sclerosis, where iron accumulates due to intrinsic dysregulation of iron metabolism in the brain (Weiland et al., 2019; Zhao et al., 2023).

Acquisition of iron by the brain is particular since the blood–brain barrier separates the CNS from the systemic circulation and brain cells do not have direct access to blood transferrin (Tf)-bound iron. Iron must first enter the brain through microvascular endothelial cells in a multi-step transcellular pathway and an endogenous Tf synthesis is required to facilitates brain cells iron uptake via TF-receptor (TFR1)-mediated endocytosis. Yet, levels of apo-transferrin in the brain interstitium are quite low such that Tf become saturated with small amounts of iron and non-Tf bound iron (NTBI, the most toxic iron form) appears as the main source of iron delivery to neural cells. By comparison to systemic NTBI that is transported in the liver by the metal-ion transporter ZIP14 a member of ZIP (Zrt- and Irt-like Protein) family, handling of NTBI into brain cells is believed to be mediated by divalent metal transporter 1 (DMT-1), although ZIP14 is also expressed in the brain. Iron storage in brain cells is classily mediated by intracellular ferritin cores. Abnormalities in iron metabolism regulation, such as iron overload or dysregulation of proteins involved in iron management (e.g., transferrin, ferritin), can increase the availability of free iron within the cell. This free iron, not bound to proteins, is particularly reactive and actively participates in Fenton reactions, which produce highly reactive hydroxyl radicals (OH•) from hydrogen peroxide (H_2_O_2_), causing lipid peroxidation, damaging cellular membranes and contributing to ferroptosis.

Previous clinical investigations have revealed a notable increase in iron deposition within anatomical regions pertinent to motor function (e.g., globus pallidus) and extrinsic sensory processing (e.g., pulvinar region) in two siblings with MPSIIIB (Brady et al., 2013). These data were confirmed in the parietal cortex of MPSIIIB mice (Puy et al., 2018), which was concomitant with the onset of neuroinflammation (Villani et al., 2009) and oxidative stress in the brain (Trudel et al., 2015).

Thus, the collective evidence of cerebral iron accumulation indicates that iron-associated neuropathology may serve as additional factor implicated in the neurodegenerative process of MPSIII.

In this article, we aim to further elucidate eventual presence of ferroptosis mechanisms in the murine model for type B of Sanfilippo Syndrome.

## 2 Materials and methods

### 2.1 Animal model

C57Bl/6 Naglu−/− (MPSIIIB) mice were purchased from Jax Lab (stain ref #003827). This model was initially obtained by introducing a neomycin resistance cassette into the exon 6 of *Naglu* gene, interrupting therefore the production of full length active NAGLU enzyme (Li et al., 1999). The experiments were conducted in male and female wildtype (WT) and MPSIIIB mice, ensuring that we included an equal number of males and females in each experiment whenever possible. They were fed standard laboratory chow for 2 or 9 months, with food and water provided *ad libitum*. All experiments were performed in accordance with European Committee Standards concerning the care and use of laboratory animals and were approved by the local ethical committee (n°122) and by the French Ministry of Higher Education and Research (n° 2020273122248406V4).

### 2.2 Mouse brain tissue processing

The mice were first subjected to euthanasia by injection of 100 mg/kg ketamine and 10 mg/kg xylazine. Blood samples were then collected by cardiac puncture, and brains were removed after heart perfusion with PBS. Isolated brains, were either harvested and frozen at −80°C for further analysis, or immediately processed for immunohistochemistry processing.

### 2.3 Brain sections immunohistochemistry

Brain samples were embedded in paraffin and sectioned into 5 µm slices. The brain sections were then deparaffinized and rehydrated using histoclearII® baths and antigen retrieval was performed using microwave heated 1x citrate buffer. The sections were then incubated with blocking solution (HRP008DAB, Zytomed) and with peroxide block solution (E41-100, Diagomics) to avoid non-specific binding of antibodies or of 3,3-diaminobenzidine (DAB) staining. Primary antibodies were used at 1:400 dilutions for anti-FPN (P1-21502, Novus Biological) and anti-FTH (DF6278, Affinity Biotech). The immunoreactive staining were revealed with the streptadivin-HRP conjugated secondary antibodies and DAB solution.

### 2.4 Tissue iron quantification

100 mg of these brain tissues were cut into 4 to 6 pieces using a non-metallic scalpel. They were placed in a microtube washed with 0.5 M HCl. After adding 400 µL of protein precipitation solution (H20, trichloroacetic acid, HCl C°36%–38%, Thioglycolic acid), the samples were placed in a double boiler at 65°C for at least 24 h. Once the liquid supernatant had been recovered and weighed, the equilibration solution (sodium acetate pH4.5) diluted 1:10 was added. The concentration of ferric iron, including free and bound iron, was then measured using an automated system (Kit Iron #SI8330, Ferrozine, Rendox Daytona+, Randox Laboratories, France).

### 2.5 RNA extraction and reverse transcriptase-quantitative polymerase chain reaction (RT-qPCR)

Total RNA was isolated using the SV Total RNA Isolation System Kit (Z3103, Promega) according to the supplier’s recommendations. The reverse transcription reaction was performed using 1.5 µg of total RNA in a final volume of 20 μL. qPCR was performed in duplicate using the SsoFast EvaGreeen PCR-mix (1725204, BioRad) and specific primers for TLR4, ZIP14, iNOS, nNOS, SIRT3, SOD2, ACSL4, LPCAT3 and ARPO transcripts (Table 1). Results were normalised to transcripts of the brain specific ARPO housekeeping gene. Results were expressed in arbitrary units as a fold change relative to the control sample using a 2^−ΔΔCT^ calculation (ΔΔCt = ΔCt exposed − mean ΔCt control). qPCR and statistical analyses were performed using StepOne Software v2.3 and PRISM (Prism 10 Version 10.1.1).

**TABLE 1:** Primers used for RT-qPCR amplifications of genes.

Gene	Forward primer	Reverse primer
ARPO	TCCAGAGGCACCATTGAAATT	TCGCTGGCTCCACCTT
NNOS	CGGGAATCAGGAGTTGCAGT	CCTCCAGCCGTTCAATGAGT
INOS	CCCTTCAATGGTTGGTACATGG	ACATTGATCTCCGTGACAGCC
SIRT3	CGCTAAACTTCTCCCGGGTT	ACACAGAGGGATATGGGCCT
SOD2	GCGGCCTACGTGAACAATCT	ATATGTCCCCCACCATTGAACT
ACSL4	ACTGGCGATATTGGAGAAT	CACATAGGACTGGTCACTT
LPCAT3	TCGTGCTTCAGTTCCTCATC	CCGGTGGCTGTGTAGTAATATC

### 2.6 Proteins extraction and western blotting (WB)

Brain tissue samples were homogenized in lysis buffer containing 250 mM sucrose, 30 mM histidine, 1x inhibitor cocktail and 0.1 mM 4-2-aminoethylbenzenesulfonyl fluoride (AEBSF), using an ultraturax at 4°C. Membrane proteins were purified by an initial centrifugation of the homogenate (2,200 rpm, 5 min at 4°C), and second ultracentrifugation (45,000 rpm for 1 h at 4°C) of the supernatant. The resulting pellet, was resuspended using a 27G needle and protein assay was performed using the Pierce TM Rapid Gold BCA Protein Assay kit according to the supplier’s recommendations (A53225, ThermoFisher). For Western blot analysis, 30 µg of proteins was separated on 4%–15% SDS-polyacrylamide pre-cast gels (Mini-PROTEAN® TGX™ Precast Gels, BioRad) and transferred to nitrocellulose membranes using the Trans-Blot® Turbo™ Transfer System (1704150, BioRad). Primary antibodies were used at 1:2000 dilutions for anti-FPN (#NBP1-21502SS, Novus Biologicals Europe, Abingdon, United Kingdom) and 1:1000 for the following antibodies: anti-FTH (#A19544, ABclonal, Düsseldorf, Germany), anti- xc- (#DF12509, Affinity Bioscience, Europe), anti-NRF2 (#AF0639, Affinity Bioscience, Europe), anti-GPX4 (#A11243, ABclonal, Düsseldorf, Germany), anti-Keap1 (#AF5266, Affinity Bioscience, Europe), anti-SOD2 (#AF5144, Affinity Bioscience, Europe). Finally, to reveal the immunolabelled proteins, the luminol-peroxidase solution (#RPN2232, Cytiva) was deposited on the membrane before being observed on the ChemiDoc Imaging System XRS + sampler (#1708265, BioRad). The results are standardized by dividing the value of the density of the band of interest over the value of the total proteins in the same sample (Supplementary Figure S1). Analyses were carried out using ImageJ software (ImajeJ2 version 2.14.0/1.54F). The complete immunoblot images presented in this article, along with their corresponding total protein staining, have been submitted to the journal for standard verification.

### 2.7 Statistical analysis

For all samples, we performed Mann-Whitney tests, which is a non-parametric test using GraphPad Prism software (Prism 10 Version 10.1.1) setting the statistical significance threshold at p < 0.05. Values were expressed as mean ± SEM.

Normalized quantification data both for WB and RTqPCR and reported in Supplementary Tables.

## 3 Results

Ferroptosis is a regulated form of cell death characterized by the accumulation of iron and lipid peroxides. Several markers and characteristics are used to identify the presence of ferroptosis in cells. The main ones are accumulation of iron, accumulation of lipid Peroxides highlighted by overexpression of ACSL4 (long-chain acyl-CoA synthetase 4) and LPCAT3 (lysophosphatidylcholine acyltransferase 3) genes, molecular and enzymatic markers such as reduced GPX4 (Glutathione Peroxidase 4), a key enzyme that protects against lipid peroxidation, decreased SLC7A11 (xc^−^), the subunit of the cystine/glutamate antiporter system that regulates cystine import for glutathione synthesis, and finally induced anti-oxidant systems including SOD (superoxide dismutase), SIRT3 (sirtuin 3), NRF2 (Nuclear Factor Erythroid 2-Related Factor 2). We therefore studied all these markers in MPSIIIB by comparison to WT mice at 2 and 9 months of age.

### 3.1 Iron accumulation of MPSIIIB brain

**FIGURE 1:**
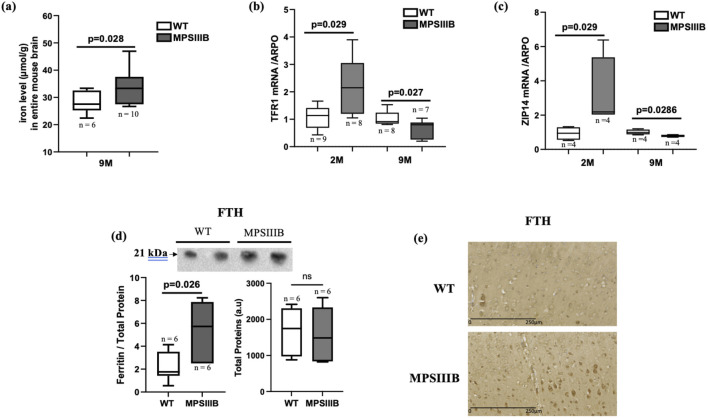
Iron parameters in the brain of MPSIIIB mice. **(A)** Measurement of iron levels in the total brain of 9 months-old WT and MPSIIIB mice. **(B, C)** mRNA abundance quantification of iron importers Transporters TFR1 **(B)** and ZIP14 **(C)** by RT-qPCR. Analyses were conducted at 2 months (2 M) and at 9 months (9 M). **(D)** Quantification of ferritin FTH protein abundance by Western blot: top, representative image of the blots; bottom left, quantification of FTH relative to total proteins; bottom right, quantification of total proteins alone. Data are quoted as the mean ± SEM. Loading blot is in Supplementary Data. **(E)** immunostaining of FTH in WT (upper image) and MPSIIIB (lower image). n ranges from 6 to 10.

We have previously demonstrated that total iron content progressively accumulates in the MPSIIIB mouse brain, becoming significant by 9 months of age. This accumulation was particularly pronounced in the cortex region. Figure 1A presents the quantification of iron in the total brain at 9 months. The results indicated that the increase in iron content was still significant compared to the WT brain, although the magnitude of this increase was smaller compared to the cortex (Figure 1A and (Puy et al., 2018)).

Furthermore, we explored the mRNA expression levels of transporters facilitating iron import into cells, TFR1 and ZIP14. We observed a significant increase in the abundance of TFR1 mRNA and an almost significant increase of ZIP14 mRNA levels in 2-month-old MPSIIIB mice compared to age-matched WT mice (Figures 1B, C). However, at 9 months, the gene expressions of the two transporters were significantly decreased mice in the MPSIIIB mouse model in comparison to WT (Figures 1B, C), evoking negative feedback on these transporters in the regard of high iron deposit within brain cells.

In response to an increase in intracellular iron, the protein level of ferritin (FTH, responsible for intracellular iron storage) rises. As expected, we observed an increase in the abundance of the protein abundance de FTH in MPSIIIB, as indicated by Western blot quantifications (Figure 1D). However, analysis of FTH staining by immunohistochemistry revealed that only a few brain cells are affected by the increased levels of this protein, suggesting a specific cell iron deposit in MPSIIIB brain (Figure 1E).

### 3.2 Accumulation of lipid peroxides

ACSL4 and LPCAT3 play critical roles in the process of ferroptosis because they facilitate the incorporation of polyunsaturated fatty acids (PUFAs) into membrane phospholipids, which increases the cell susceptibility to lipid peroxidation (Gan B., 2021). In ferroptosis, both ACSL4 and LPCAT3 are upregulated (Doll et al., 2017; Reed et al., 2022).

**FIGURE 2:**
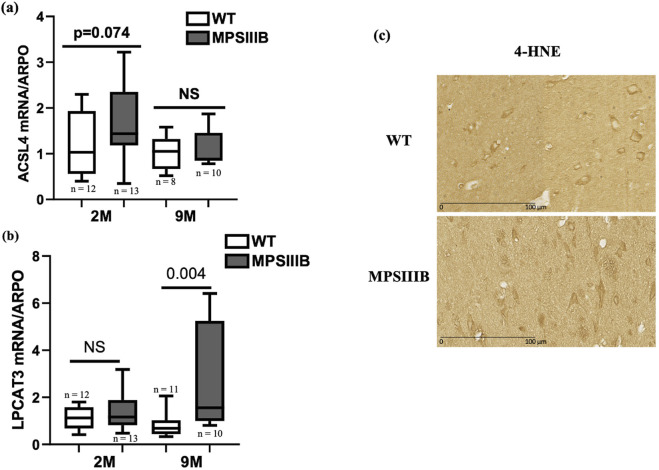
The lipid peroxidation process involved in the MPSIIIB model. Analyses of mRNA expressions of ACSL4 **(A)** and LPCAT3 **(B)** markers of lipid peroxidation by RT-qPCR in WT (white bars) and MPSIIIB (gray bars) mice aged 2 and 9 months. Data are quoted as the mean ± SEM. n ranges from 9 to 15. **(C)** Immunostaining of 4-HNE in WT (upper image) and MPSIIIB (lower image).

At 2 months of age, MPSIIIB mice exhibited a modest increase in RNA levels for both ACSL4 and LPCAT3 genes compared to WT mice (Figures 2A, B). However, LPCAT3 mRNA showed a pronounced increase in 9-month-old MPSIIIB mice (Figure 2B). To avoid missing the temporal dynamics of ACSL4 induction in MPSIIIB, we additionally assessed the level of its mRNA expression at 5 months of age, revealing a significant elevation in MPSIIIB brains compared to WT brains (data not shown).

4-HNE is a reactive compound generated by the oxidative breakdown of polyunsaturated lipids, a process frequently observed during ferroptosis. Its accumulation is a key indicator of the intensity of lipid peroxidation. Immunohistochemical analysis revealed a significant increase in the 4-HNE marker in cells from the brains of 9-month-old MPSIIIB mice, compared with those from the brains of WT mice of the same age (Figure 2C).

### 3.3 Activation of oxidative stress pathways

**FIGURE 3:**
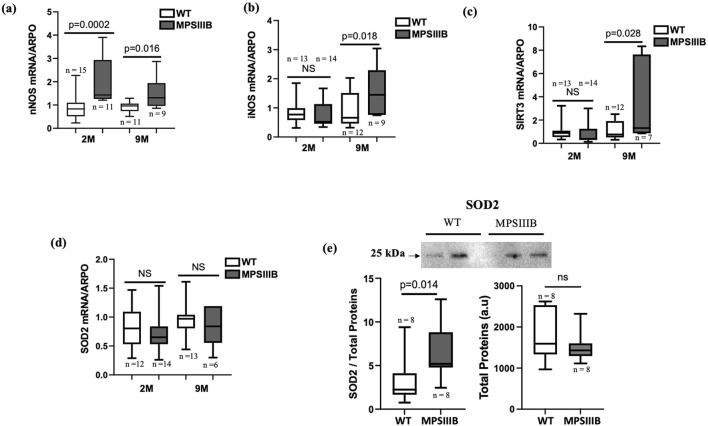
Oxidative stress imbalance in the MPSIIIB model. Analysis of oxidative stress parameters in WT mice and MPSIIIB mice aged 2 and 9 months. nNOS **(A)** and iNOS **(B)** are the specific pro-oxidant markers. SIRT3 **(C)** and SOD2 **(D)** are the antioxidant markers analyzed by RT-qPCR. **(E)** Quantification of SOD2 protein abundance by Western blot: top, representative image of the blots; bottom left, quantification of SOD2 relative to total proteins; bottom right, quantification of total proteins alone. Data are quoted as the mean ± SEM. Loading blot is in Supplementary Data. n ranges 7 to 16 mice.

Given that we have already observed an elevation of ROS levels in the MPSIIIB model (Villani et al., 2009; Trudel et al., 2015), we opted to investigate the expression of both pro- and anti-oxidant genes in the MPSIIIB brain. For pro-oxidant genes, the mRNA expression of neuronal nitric oxide synthase (nNOS), the constitutive form, was significantly elevated in the MPSIIIB brain at both 2 and 9 months of age, and the mRNA levels of inducible nitric oxide synthase (iNOS) showed a significant increase at 9 months of age (Figures 3A, B). However, the expression levels of the NADPH oxidases NOX2 and NOX4, responsible for the production of superoxide and hydrogen peroxide, remained unchanged in both WT and MPSIIIB mice (Supplementary Figure S2). For the anti-oxidant genes, we observed that the mRNA expression level of SIRT3 is significantly increased in MPSIIIB mice compared to WT mice at both ages (Figure 3C). The mRNA levels of the antioxidant SOD2 (Superoxide Dismutase 2), tended to increase at 9 months for MPSIIIB mice, however, when we explored its protein abundance, we observed SOD2 highly increased in 9-month-old MPSIIIB compared to WT mice (Figures 3D, E). This difference can be explained by post-transcriptional regulation or mechanisms that increase the stability of the SOD2 protein.

**FIGURE 4:**
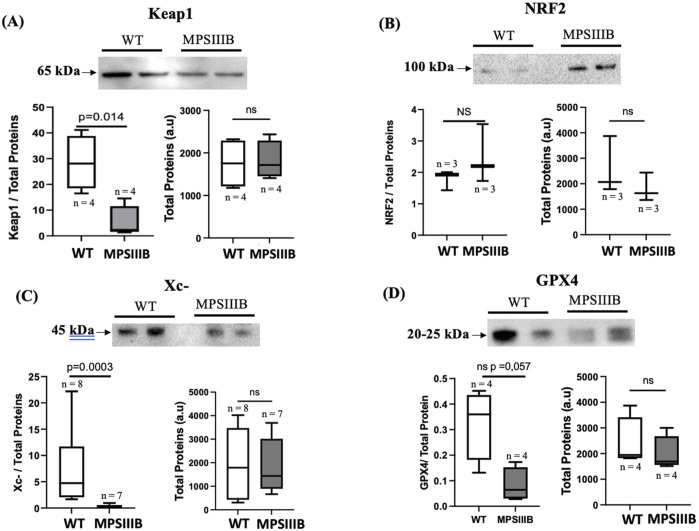
Activation of the Keap1/NRF2 and disregulation of the Xc-/gpx4 system in BMPIIIB model. (A) Analysis were made by Western blot in 9 months-old WT and MPSIIIB mice. Top images are representative blots; bottom left, are quantifications of Keap1 **(A)**, NRF2 **(B)**, Xc- **(C)** and GPX4 **(D)** relative to total proteins; bottom right, quantification of total proteins alone. Data are quoted as the mean ± SEM. Loading blot is in Supplementary Data. n ranges 3 to 8 mice.

Keap1 (Kelch-like ECH-associated protein 1) is a key regulatory protein involved in the oxidative stress response and is primarily known for its role in controlling the activity of the transcription factor Nrf2 (Nuclear factor erythroid 2-related factor 2). The regulation of Keap1 during ferroptosis, occurs through multiple mechanisms including its protein degradation (Sun et al., 2016). Protein analysis of the brains of 9-month-old mice (Figures 4A, B) revealed significantly decrease of Keap1 in MPSIIIB compared to their WT littermate. The total NRF2 protein level remained unchanged, which may be expected because NRF2 is activated by translocation to the nucleus, where its abundance should be enhanced.

The Xc^−^ system and GPX4 (glutathione peroxidase 4) are critical components of the cellular defense mechanism against oxidative stress. In ferroptosis, disruption of the Xc^−^/GPX4 axis is a hallmark of induced ROS and PUFA peroxidation. In the brain of MPSIIIB mice, WB experiments (Figure 4C), showed that the protein expression of the Xc-antiporter is significantly reduced in 9-month-old MPSIIIB mice compared to WT mice. In addition, GPX4 protein expression was also decreased in MPSIIIB compared WT brains (Figure 4D), confirming promotion of ferroptosis in MPSIIIB.

### 3.4 MPSIIIB-FPN distribution and brain cells affected by ferroptosis

**FIGURE 5:**
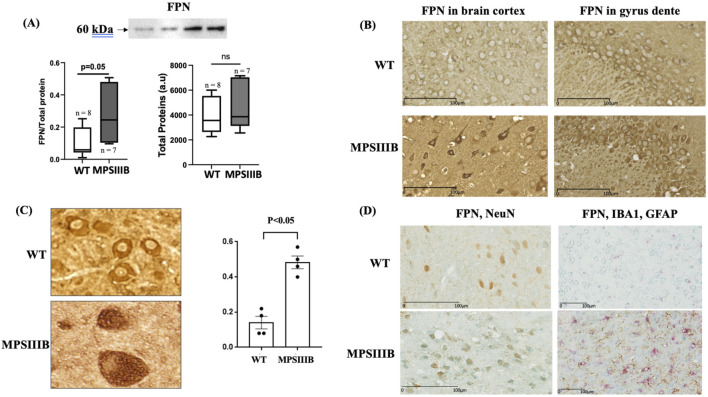
Misfolding of ferroportin (FPN) specifically in neurons. Analysis were performed in 9 months-old WT and MPSIIIB mice. **(A)** Quantification of FPN protein abundance by Western blot: top, representative image of the blots; bottom left, quantification of FPN relative to total proteins; bottom right, quantification of total proteins alone. Data are quoted as the mean ± SEM. Loading blot is in Supplementary Data. **(B)** Labeling of FPN on brain sections. Representative images were taken in cortex (left panel) and gyrus dente (right panel). **(C)** Quantification of brain cells with vacuolar FPN in WT and MPSIIIB. Data are quoted as the mean ± SEM. **(D)** Co-immunolabeling of brain sections with FPN (Blue) and NeuN (brown) (left panel) and with FPN (Blue), IBA1 (PINK) and GFAP (Brown) (right panel). The accumulation of FPN is specifically present in NeuN-marked cells, the neurons.

Ferroportin (FPN), involved in cellular iron export, is also assumed to be increased at protein level in response to augmented intracellular iron. Similarly to FTH, data from WB analysis (Figure 5A) and immunostaining (Figure 5B) also showed that the abondance of FPN was increased in MPSIIIB brain. Of interest, the results of FPN distribution in MPSIIIB, revealed that the increase of the protein was mainly intracellular with only a small amount of the protein located at the cell membrane (Figures 5B, C). The retention of FPN in theses vacuoles was threefold increase in MPSIIIB mice compared to WT cells (Figure 5B), but the origin of these cellular vacuoles remained to be determined. We took advantage of the cell-specific distribution of FPN in MPSIIIB brain to identify cerebral cells affected by this increase, which may indicate specific cell iron accumulation and ferroptosis. We therefore performed co-localization experiments by immunolabeling FPN and markers of the three MPSIIIB-affected cells, microglia, astrocytes and neurons. The results indicated a high colocalization of FPN with the nuclear protein NeuN, a specific marker of neurons. In cells expressing Iba1 (microglia) or GFAP (astrocytes), there was no increase in FPN abundance nor retention of the protein, suggesting a neuron-specific alteration (Figure 5D).

## 4 Discussion

This study provides compelling evidence for the presence of ferroptosis as pathogenesis in MPSIIIB mice, and highlighted the neuron as the first target of specific accumulation of iron.

The observed increase in iron content in the total brain of adult MPSIIIB mice aligns with our previous study showing increased iron content in the parietal cortex of MPSIIIB mice and with data from patients evidencing iron deposits in specific region of brain by MRI (Brady et al., 2013), reinforcing the notion that iron dysregulation is a significant feature of MPSIIIB disorder. In addition, the findings indicating a more modest overall increase in iron content in the total brains, compared to the isolated parietal cortex of MPSIIIB mice, suggest that the extent of iron accumulation may be restricted rather than widespread across all brain regions. This is may be due to the fact that not all brain cell populations are equally affected by iron retention. Indeed, misfolding of the iron exporter FPN was colocalized only with neurons. Thus, we hypothesize only neurons must be concerned with ferroptosis in MPSIIIB.

The mechanism leading to iron accumulation is not fully elucidated, but our current data may suggest a plausible pathway: 1- Iron uptake appears to begin in early adulthood (2 months of age), according to an increase in the expression of transferrin receptor 1 (TFR1) and ZIP14 which are responsible for the transport of transferrin-bound and non-transferrin-bound iron (NTBI) iron, respectively. However, TFR1 and ZIP14, may not be the only involved in the iron accumulation in neurons, as both genes are significantly downregulated in aged mice (9 months). This downregulation is likely mediated by the iron response element (IRE)/iron regulatory protein (IRP) system in response to cellular iron overload. IRE sequences are located in the 3′UTR of the iron importer TFR1 and in the 5′UTR of iron sequestrators and exporters (FTH, FTL, and FPN). Under low intracellular iron conditions, IRE-IRP binding stabilizes TFR1 mRNA, while reducing the translation of FTH, FTL, and FPN encoding mRNAs. Conversely, under high iron conditions, the reduced binding leads to increase the protein levels of FTH, FTL, and FPN, and decreased the mRNA Level of TFR1. However, the regulation of ZIP14 does not appear to be iron-dependent (Bogdan et al., 2016), and its regulatory mechanisms in the context of MPSIIIB remain to be explored. Interestingly, Muller et al. reported that the glycoprotein CD44, found on the surface of various cells in different organs, acts as a novel molecular player mediating toxic iron entry by internalizing iron bound to hyaluronic acid (Muller et al., 2020). CD44 is also expressed in neurons and may facilitate iron import, especially if heparan sulfate oligosaccharides (HSOs), which are highly generated in the brains of MPSIIIB mice, can bind iron similarly to hyaluronic acid. 2- The intracellular iron-sequestering protein ferritin was elevated at both 2 and 9 months. While ferritin levels can increase in response to iron accumulation, they can also be elevated due to inflammation, independently to iron levels. However, we have already shown that neuroinflammation started in MPSIIIB mice at the early stage (<2 months) and at 9 months, the inflammatory cytokines were only at low levels. In addition, the results from immunostaining, showed higher ferritin levels in neurons but not in inflammatory microglia or astrocytes (data not shown). Therefore, the increase in ferritin is likely a response to iron accumulation. 3- Finally, ferroportin (FPN), the only identified iron exporter to date, was upregulated, confirming its response to increased intracellular iron levels. However, immunostaining results indicated retention of FPN intracellularly, which may lead to impaired iron export and subsequent accumulation in neurons. The vacuoles that retained FPN protein, may represent defective lysosomes, given that MPSIIIB is a lysosomal storage disease and FPN is a target for trafficking to lysosomes (Nemeth et al., 2004). We also hypothesize that a defect in FPN ubiquitination contributes to its retention and accumulation. This hypothesis aligns with previous findings in MPSIIIB. For instance, a study on fibroblasts from MPSIIIB patients reported reduced levels of ubiquitinated proteins, suggesting impaired proteasomal activity, which may contribute to the accumulation of misfolded FPN in this disorder (Pierzynowska et al., 2020). Additionally, proteomic analysis of mouse brains revealed dysregulation of cytoskeletal and metabolic proteins, along with dysfunction in protein degradation pathways (Leal et al., 2023; Rintz et al., 2023), including the ubiquitin-proteasome system and autophagy, leading to the accumulation of misfolded proteins (De Pasquale et al., 2020). Previous studies have also reported alterations in p62 and LC3-B levels in MPSIIIB, indicating tissue-specific regulation of autophagy and a potential blockage of autophagic flux at the lysosomal level. Further research is needed to elucidate these mechanisms, particularly in relation to FPN retention and iron accumulation in this context (De Pasquale et al., 2020; Pierzynowska et al., 2020; Schiattarella et al., 2015).

Intracellular iron accumulation leads to the production of reactive oxygen species (ROS), which is consistent with the induction of nNOS and iNOS observed in the MPSIIIB brain. nNOS is a soluble enzyme constitutively expressed in neurons; however, its induction in the context of MPSIIIB may be detrimental, as elevated nNOS levels have been associated with cytosolic toxicity and neuronal death (Dawson et al., 1998; Dias et al., 2024). In contrast, iNOS is an inducible enzyme, and its activation was expected due to the inflammatory environment of MPSIIIB (Martin et al., 2015; Puy et al., 2018).

Regarding ferroptosis, several biomarkers support the presence of this iron-dependent neuronal death in MPSIIIB. In addition to the hallmark reduction in xc^−^/GPX4, which characterizes ferroptosis, we observed increased levels of antioxidants such as mitochondrial SIRT3 and SOD2. These antioxidants enhance the activity of ROS-eliminating enzymes. The Keap1/NRF2 system, a key indicator of ferroptotic signaling (Kansanen et al., 2013; Kim et al., 2020) was also induced, further supporting the ferroptotic profile. Moreover, increased lipid peroxidation was highlighted by the upregulation of ACSL4 and LPCAT3 genes. ACSL4 initiates lipid peroxidation, while LPCAT3 sensitizes lipids to this process. In MPSIIIB model, ACSL4 showed a tendency towards increased expression (although this did not reach statistical significance), while LPCAT3 was significantly upregulated in aging mice. This pattern is consistent with the temporal dynamics of lipid peroxidation, where ACSL4 is activated earlier than LPCAT3. Our findings align with this progression, with increased sensitivity to ACSL4 likely before 2 months and to LPCAT3 at 9 months of age.

In conclusion, our data demonstrate the presence of neuronal iron dyshomeostasis and ferroptosis in the MPSIIIB model. These findings, coupled with previous reports from MPSIIIB patients (Brady et al., 2013), suggest that iron accumulation plays a significant role in the progression of neurodegenerative forms of MPSIII. This highlights the need to explore potential advancing therapeutic strategies that incorporate selective inhibitors of iron chelation and ferroptosis. Assessing the efficacy of these inhibitors may be beneficial in conjunction with upcoming gene therapy clinical trials, as well as providing alternative treatment options for patients who are not eligible for potential gene therapies.

## Data Availability

The original contributions presented in the study are included in the article/Supplementary Material, further inquiries can be directed to the corresponding author.

## Supplementary material

SUPPLEMENTARY FIGURE S1 Normalization of protein loading using the Stain-Free gel system. Representative Stain-Free gel images showing total protein loading for each sample. A consistent amount of 30 µg of protein was loaded per lane. Normalization was performed using ImageJ by correlating the intensity of bands of interest to the total protein signal. **(a)** FTH loading blot. (SOD2) FTH loading blot. **(c)** Keap1 loading blot. **(d)** NRF2 loading blot. **(e)** Xc- loading blot. **(f)** GPX4 loading blot. **(g)** FPN loading blot.

SUPPLEMENTARY FIGURE S2 Analyses of mRNA expressions of NOX2 **(A)** and NOX4 **(B)** markers of oxidant stress by RT-qPCR in WT (white bars) and MPSIIIB (gray bars) mice aged 2 and 9 months. Data are quoted as the mean ± SEM. n ranges from 3 to 13.
