# Supplementary figures and images for: Identification of a neuron-specific ferroptosis in the neurodegenerative mucopolysaccharidosis III model
Source: Front Mol Biosci. 2025 Mar 18;12:1476513. doi: 10.3389/fmolb.2025.1476513 (PMC11959000; doi:10.3389/fmolb.2025.1476513)

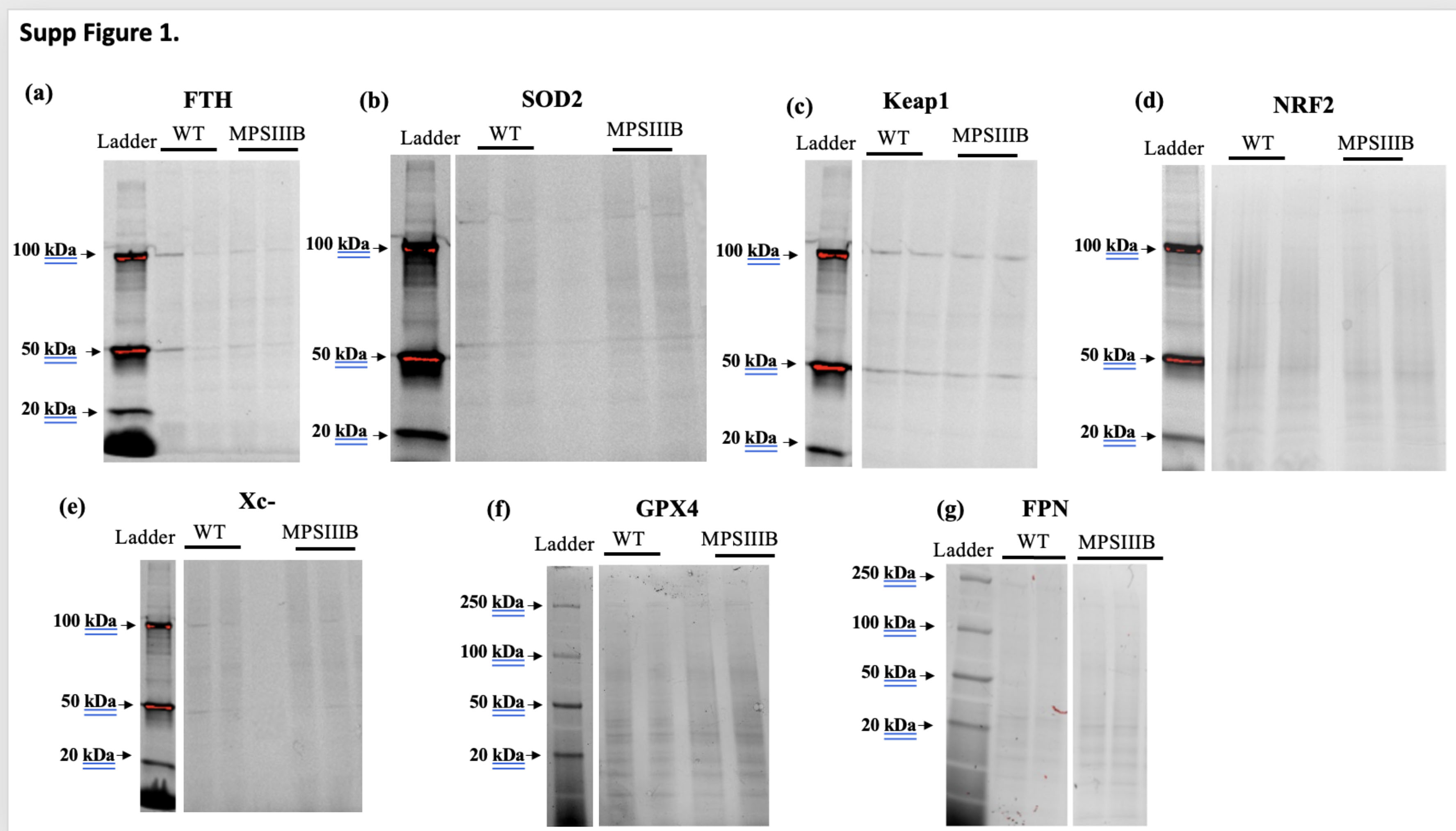

Supplement: Supplementary file 1 [file Image1.jpeg]

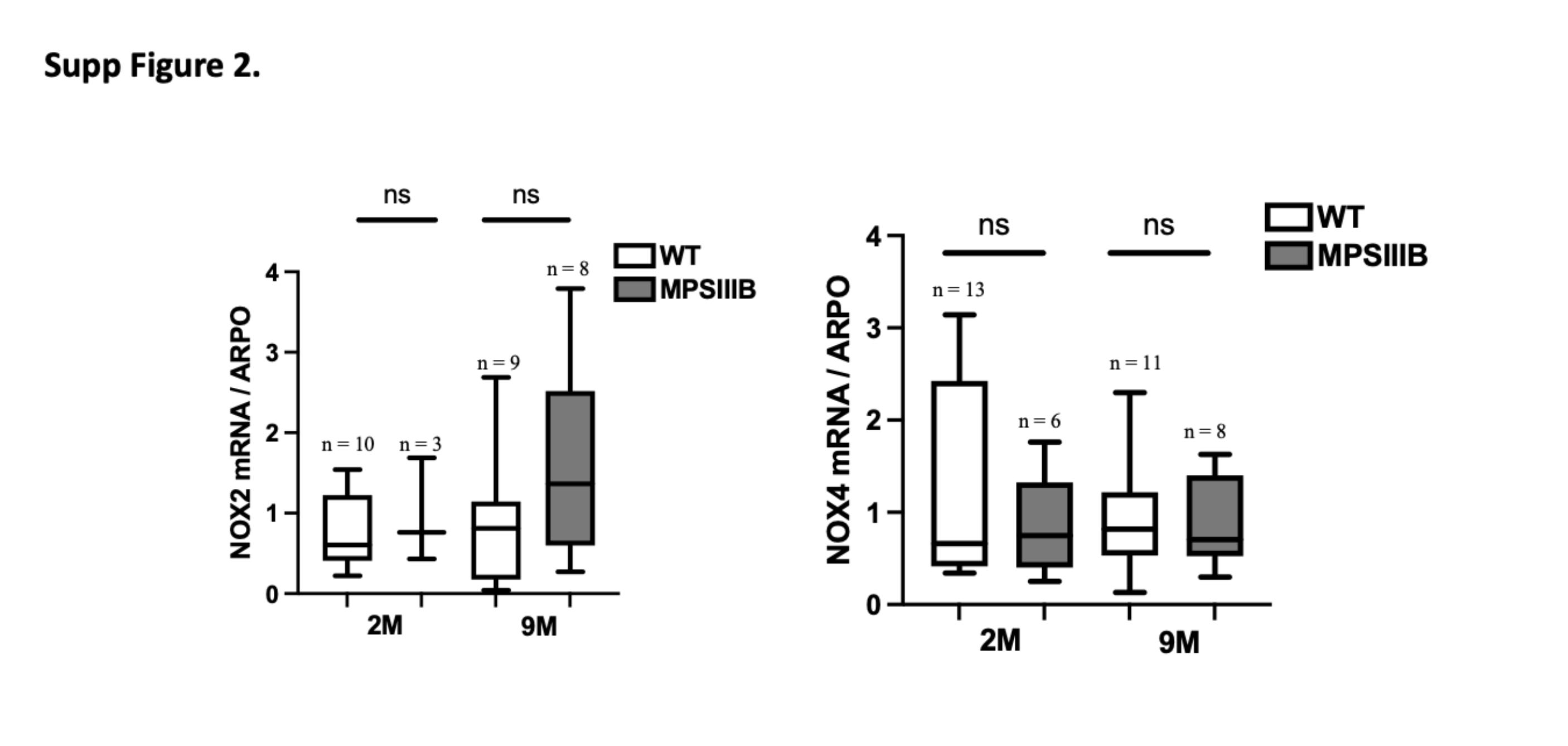

Supplement: Supplementary file 2 [file Image2.jpeg]
