# Supplementary material for: Identification of a neuron-specific ferroptosis in the neurodegenerative mucopolysaccharidosis III model
Source: Front Mol Biosci. 2025 Mar 18;12:1476513. doi: 10.3389/fmolb.2025.1476513 (PMC11959000; doi:10.3389/fmolb.2025.1476513)
Supplement: Supplementary file 3 [file DataSheet1.pdf]

Supp TABLE 1. Quantification data from RT-qPCR (2months)

nNOS		
**Samples**	**Genotype**	**Normalisation (2-ΔΔCt)**
1	**WT**	1,00
2		1,12
3		1,07
4		0,65
5		0,23
6		0,46
7		1,09
8		0,92
9		2,17
10		2,27
11		0,74
12		0,54
13		0,74
14		2,96
15		0,25
**Samples**	**Genotype**	**Normalisation (2-ΔΔCt)**
1	**MPSIIIB**	3,90
2		1,53
3		2,96
4		1,26
5		1,26
6		1,43
7		2,93
8		2,79
9		1,21
10		1,34
11		1,24
**iNOS**		
**Samples**	**Genotype**	**Normalisation (2-ΔΔCt)**
1	**WT**	1,61
2		0,81
3		0,31
4		0,50
5		0,75
6		1,85
7		0,90
8		0,77
9		1,08
10		0,44
11		0,69
12		0,77
13		0,66
**Samples**	**Genotype**	**Normalisation (2-ΔΔCt)**
1	**MPSIIIB**	0,50
2		0,37
3		1,21
4		0,50
5		0,53
6		0,55
7		0,52

8		1,26
9		1,67
10		2,36
11		0,88
12		2,59
13		0,44
14		0,34
**SIRT3**		
**Samples**	**Genotype**	**Normalisation (2-ΔΔCt)**
1	**WT**	1,10
2		0,85
3		0,65
4		0,33
5		0,58
6		0,54
7		1,05
8		0,48
9		1,34
10		1,08
11		0,52
12		0,93
13		1,04
**Samples**	**Genotype**	**Normalisation (2-ΔΔCt)**
1	**MPSIIIB**	3,00
2		0,38
3		0,28
4		0,37
5		0,13
6		0,38
7		0,30
8		1,42
9		0,48
10		0,73
11		0,62
12		0,38
13		0,29
14		0,26
**SOD2**		
**Samples**	**Genotype**	**Normalisation (2-ΔΔCt)**
1	**WT**	1,47
2		0,75
3		0,50
4		0,29
5		0,54
6		0,86
7		0,91
8		1,04
9		1,25
10		0,75
11		0,45
12		0,70
**Samples**	**Genotype**	**Normalisation (2-ΔΔCt)**
1	**MPSIIIB**	0,62
2		0,53
3		0,67

4		0,69
5		0,53
6		0,55
7		0,66
8		1,50
9		0,64
10		1,54
11		1,01
12		0,78
13		0,30
14		0,26
**NOX2**		
**Samples**	**Genotype**	**Normalisation (2-ΔΔCt)**
1	**WT**	1,45
2		0,62
3		0,59
4		0,22
5		0,35
6		0,43
7		0,72
8		1,15
9		1,54
10		0,47
**Samples**	**Genotype**	**Normalisation (2-ΔΔCt)**
1	**MPSIIIB**	1,69
2		0,43
3		0,76
**NOX4**		
**Samples**	**Genotype**	**Normalisation (2-ΔΔCt)**
1	**WT**	3,08
2		0,70
3		0,34
4		3,14
5		0,41
6		0,66
7		0,75
8		2,76
9		0,47
10		0,36
11		0,42
12		2,09
13		0,51
**Samples**	**Genotype**	**Normalisation (2-ΔΔCt)**
1	**MPSIIIB**	0,63
2		0,25
3		1,76
4		0,87
5		1,18
6		0,45
**ACSL4**		
**Samples**	**Genotype**	**Normalisation (2-ΔΔCt)**
1	**WT**	1,15
2		1,18
3		0,94
4		2,3

5		2,3
6		0,93
7		0,41
8		2,3
9		0,4
10		1,12
11		2,18
12		0,84
**Samples**	**Genotype**	**Normalisation (2-ΔΔCt)**
1	**MPSIIIB**	2,39
2		1,44
3		2,31
4		1,27
5		1,42
6		1,46
7		1,49
8		0,35
9		2,73
10		0,67
11		1,1
12		3,22
13		1,28
**LPCAT3**		
**Samples**	**Genotype**	**Normalisation (2-ΔΔCt)**
1	**WT**	0,65
2		1,16
3		1,12
4		0,77
5		1,48
6		1,80
7		0,45
8		1,12
9		1,79
10		0,42
11		1,61
12		0,95
**Samples**	**Genotype**	**Normalisation (2-ΔΔCt)**
1	**MPSIIIB**	0,83
2		2,02
3		1,38
4		1,55
5		0,83
6		0,85
7		1,80
8		0,81
9		1,84
10		0,48
11		0,95
12		2,77
13		0,74
14		3,18

Supp TABLE 3. Quantification data from RT-qPCR (9months)

**nNOS**		
**Samples**	**Genotype**	**Normalisation (2-ΔΔCt)**
1	**WT**	0,51
2		0,97
3		0,53
4		0,85
5		1,06
6		1,29
7		0,76
8		1,20
9		0,96
10		0,74
11		1,04
**Samples**	**Genotype**	**Normalisation (2-ΔΔCt)**
1	**MPSIIIB**	0,95
2		1,31
3		1,18
4		0,97
5		0,86
6		2,87
7		1,66
8		2,22
9		1,42
**iNOS**		
**Samples**	**Genotype**	**Normalisation (2-ΔΔCt)**
1	**WT**	0,66
2		0,46
3		0,32
4		2,03
5		0,85
6		2,21
7		1,62
8		1,51
9		0,42
10		0,49
11		0,71
12		0,56
**Samples**	**Genotype**	**Normalisation (2-ΔΔCt)**
1	**MPSIIIB**	2,12
2		0,75
3		1,09
4		3,04
5		2,46
6		1,45
7		1,87
8		0,77
9		0,75
**SIRT3**		
**Samples**	**Genotype**	**Normalisation (2-ΔΔCt)**
1	**WT**	0,3
2		0,56
3		0,78
4		0,5

5		0,48
6		0,67
7		2,31
8		2,5
9		2,23
10		0,75
11		0,9
12		0,96
**Samples**	**Genotype**	**Normalisation (2-ΔΔCt)**
1	**MPSIIIB**	1,05
2		1,31
3		0,85
4		0,86
5		8,34
6		7,64
7		5,61
**SOD2**		
**Samples**	**Genotype**	**Normalisation (2-ΔΔCt)**
1	**WT**	0,99
2		1,61
3		1,05
4		0,82
5		1,01
6		1,14
7		0,8
8		0,44
9		0,85
10		0,95
11		0,99
12		0,77
13		1,10
**Samples**	**Genotype**	**Normalisation (2-ΔΔCt)**
1	**MPSIIIB**	0,79
2		1,19
3		8,07
4		5,62
5		0,30
6		9,39
7		10,35
8		0,81
9		0,84
**NOX2**		
**Samples**	**Genotype**	**Normalisation (2-ΔΔCt)**
1	**WT**	0,04
2		0,14
3		0,91
4		0,21
5		0,65
6		0,86
7		0,81
8		1,38
9		2,69
**Samples**	**Genotype**	**Normalisation (2-ΔΔCt)**
1	**MPSIIIB**	0,99
2		2,30

3		0,47
4		0,27
5		2,59
6		3,79
7		1,68
8		1,05
	**NOX4**	
**Samples**	**Genotype**	**Normalisation (2-ΔΔCt)**
1	**WT**	0,13
2		0,62
3		0,59
4		0,39
5		0,82
6		0,53
7		2,30
8		1,22
9		1,72
10		0,83
11		0,91
**Samples**	**Genotype**	**Normalisation (2-ΔΔCt)**
1	**MPSIIIB**	0,74
2		1,08
3		0,50
4		0,30
5		1,51
6		1,63
7		0,67
8		0,60
	**ACSL4**	
**Samples**	**Genotype**	**Normalisation (2-ΔΔCt)**
1	**WT**	1,39
2		1,12
3		0,52
4		0,87
5		0,6
6		1,58
7		1,05
8		1,05
**Samples**	**Genotype**	**Normalisation (2-ΔΔCt)**
1	**MPSIIIB**	0,88
2		0,86
3		0,88
4		0,78
5		0,85
6		3,6
7		3,53
8		1,87
9		1,46
	**LPCAT3**	
**Samples**	**Genotype**	**Normalisation (2-ΔΔCt)**
1	**WT**	1,41
2		0,77
3		0,60
4		0,56
5		2,06

6		0,39
7		0,83
8		0,90
9		0,33
10		0,45
**Samples**	**Genotype**	**Normalisation (2-ΔΔCt)**
1	**MPSIIIB**	1,03
2		1,73
3		0,90
4		0,81
5		1,04
6		6,41
7		5,15
8		5,53
9		4,50
10		1,38

Supp TABLE 4. Data from Western blots normalisation (protein of interest / total proteins)

**Ferritin**		
**Samples**	**Genotype**	**Normalisation**
1	WT	4,1355717
2		1,74878975
3		1,77054016
4		0,555311076
5		1,714498213
6		3,314769989
**Samples**	**Genotype**	**Normalisation**
1	MPSIIIB	7,7386772
2		4,48439642
3		6,21208793
4		2,501744888
5		2,502018845
6		5,249393216
**Ferroportin**		
**Samples**	**Genotype**	**Normalisation**
1	**WT**	0,01050051
2		0,25205564
3		0,04217652
4		0,19838384
5		0,05636076
6		0,05873901
7		0,11702701
8		0,22486902
**Samples**	**Genotype**	**Normalisation**
1	**MPSIIIB**	0,1060195
2		0,37548529
3		0,47191589
4		0,09646491
5		0,50703288
6		0,11281467
7		0,06607946
**XC-**		
**Samples**	**Genotype**	**Normalisation**
1	**WT**	22,2033157
2		12,5973706
3		9,06707405
4		2,24819404
5		4,93498489
6		4,93498489
7		1,98310611
8		4,54113218
**Samples**	**Genotype**	**Normalisation**
1	**MPSIIIB**	0,94135492
2		0,58132473
3		0,31664104
4		0,05329375
5		0
6		0
7		0

GPX4		
**Samples**	**Genotype**	**Normalisation**
1	**WT**	0,45197517
2		0,33261658
3		0,38701874
4		0,13147668
**Samples**	**Genotype**	**Normalisation**
1	**MPSIIIB**	0,17313662
2		0,0911655
3		0,02810763
4		0,0373384
**KEAP1**		
**Samples**	**Genotype**	**Normalisation**
1	**WT**	41,1481699
2		31,8148076
3		24,4107605
4		16,5364534
**Samples**	**Genotype**	**Normalisation**
1	**MPSIIIB**	14,5755349
2		2,44634109
3		1,36292261
4		2,14202184
**NRF2**		
**Samples**	**Genotype**	**Normalisation**
1	**WT**	1,43305638
2		1,92839993
3		2,10451864
**Samples**	**Genotype**	**Normalisation**
1	**MPSIIIB**	3,53818934
2		2,2019368
3		1,72869002
**SOD2**		
**Samples**	**Genotype**	**Normalisation**
1	**WT**	1,94553493
2		1,56714602
3		9,39130707
4		2,51128902
5		4,1540526
6		1,96866482
7		3,99575042
8		0,75709563
**Samples**	**Genotype**	**Normalisation**
1	**MPSIIIB**	9,62685325
2		12,5890963
3		4,76764416
4		5,13810265
5		4,82899731
6		6,40342582
7		2,4546588
8		5,25723633
